# Thoracic Endovascular Aortic Repair in a Patient with Mobile Aortic Thrombosis

**DOI:** 10.1155/2014/302346

**Published:** 2014-02-06

**Authors:** Graham M. Lohrmann, Ferande Peters

**Affiliations:** ^1^Chris Hani Baragwanath Academic Hospital, Old Potchefstroom Road, Soweto, Johannesburg 2013, South Africa; ^2^Flora Clinic, William Nicol Drive, Floracliffe, Johannesburg 1709, South Africa

## Abstract

A 58-year-old female presented with acute arterial insufficiency to her left leg. Following cardiovascular evaluation using multimodality imaging, it was discovered that she had mobile thoracic thrombi overlying a normal descending thoracic aorta which had also caused a splenic infarction. This patient was treated with unfractionated heparin for three days and underwent subsequent thoracic endovascular aortic repair (TEVAR) uneventfully with no subsequent complications at one-year followup. This case highlights the diagnostic and therapeutic challenges in treating patients with this uncommon challenging clinical scenario.

## 1. Case Scenario

A 58-year-old female presented with symptoms and signs compatible with an acutely threatened left leg. Her history revealed that she had no preexisting significant cardiovascular symptoms of note and she had no risk factors for atherosclerosis. There was no family history to suggest any hereditary thrombotic disorders.

**Figure 1:**
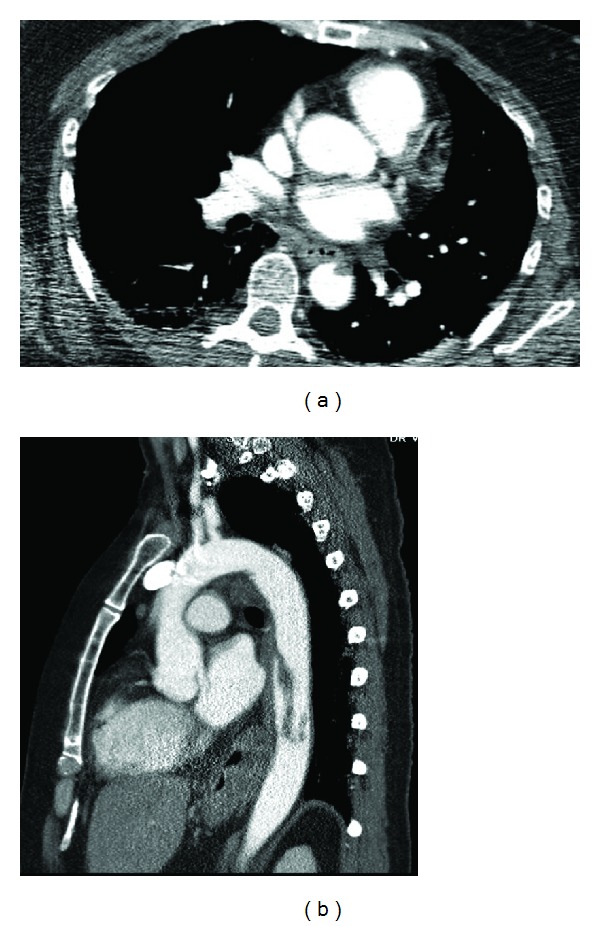
Axial and sagittal images of a computed tomography angiogram demonstrating a serpiginous filling defect in the descending thoracic aorta.

A computed tomography angiogram (CTA) was performed which confirmed abrupt cutoff of the left mid- to distal tibial artery in the left leg, as well as a recanalized splenic infarction. In addition, as part of her workup before referral, a CTA of the thoracic aorta was performed which revealed a linear filling defect measuring 8 cm which was interpreted as an aortic dissection with an intimal flap (Figures 1(a) and 1(b)).

**Figure 2:**
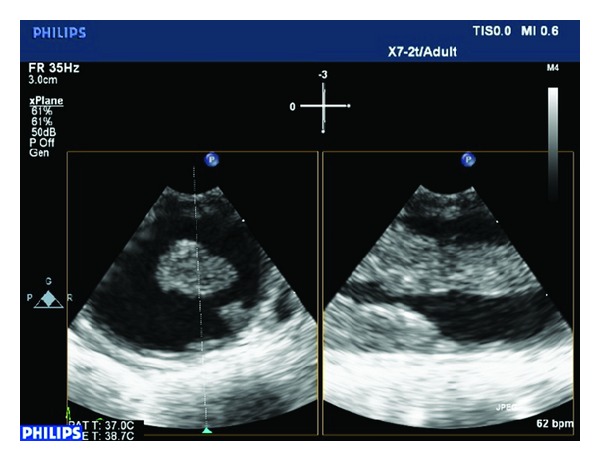
Two-dimensional transesophageal echocardiogram (2D TEE) X-plane images demonstrating two aortic thrombi.

**Figure 3:**
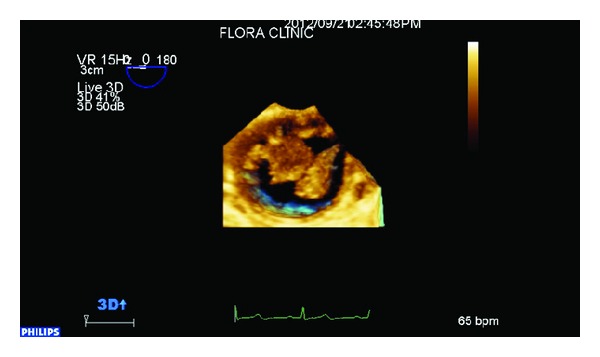
Three-dimensional transesophageal echocardiogram (3D TEE) demonstrating two thrombi in cross-section.

**Figure 4:**
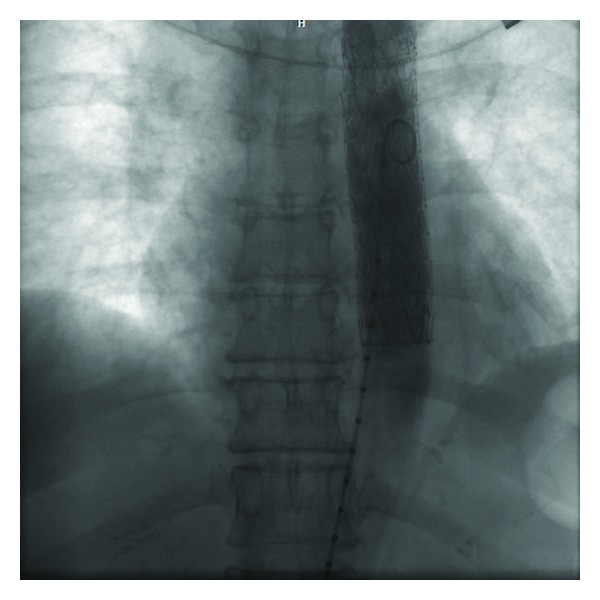
Poststent deployment aortic angiogram demonstrating successful exclusion of the thrombus bulk with a small area of residual thrombus at the inferior edge.

The patient was transferred for cardiological assessment where a transthoracic echocardiography revealed hypertensive heart disease with a normal ejection fraction and mild diastolic dysfunction. A transesophageal echo (TEE) revealed a large mobile aortic thrombus in the proximal descending aorta distal to the subclavian artery origin accompanied by two other distal mobile thrombi (Figures 2 and 3, Videos 1, 2, and 3 in see Supplementary material available online at http://dx.doi.org/10.1155/2014/302346.). There were no overt features of either a penetrating aortic ulcer or aortic dissection. The laboratory workup for hereditary and acquired prothrombotic disorders, including vasculitis, was normal. The patient was treated for three days with an intravenous unfractionated heparin infusion and, following extensive cardiothoracic and vascular consultation, thoracic endovascular aortic repair (TEVAR) was successfully performed (Figure 4), whilst the leg ischaemia resolved spontaneously on anticoagulation alone. Following initiation of lifelong warfarin therapy, a follow-up TEE six months later revealed no thrombus or subsequent complications.

## 2. Discussion

Thoracic aortic mobile thrombus (TAMT) is generally defined as mobile thrombus occurring in a nonaneurysmal thoracic aorta such that embolism is thought likely to occur from separation of the thrombus body from its base.

It is a relatively rare clinical entity with few case reports and series published since the regular use of CTA and TEE was introduced in locating arterial embolic sources. According to a large postmortem study of 10, 671 autopsies by Machleder and associates [1], the incidence of intraluminal mural thrombosis in nonaneurysmal thoracic aortas was 0.09%, with no distinction between mobile and adherent thrombi. Tunick et al., in their study of 122 patients having a TEE for suspected cardioembolic stroke, reported an aortic atheroma incidence of 27%, of which 30% had overlying TAMT [2].

TAMT is most commonly identified during a workup for possible cardioembolic clinical presentations on either CT/echocardiography. Commonly encountered cardioembolic presentations include stroke [3–6], an acutely threatened limb or blue toe syndrome [7, 8], and abdominal ischaemic syndromes [3, 9–11], including renal or splenic infarcts and mesenteric ischaemia. However, a few authors have reported asymptomatic TAMT following thoracic CTA done for unrelated reasons, including to rule out pulmonary emboli or an acute traumatic aortic syndrome [12, 13].

The underlying pathogenesis of aortic thrombosis has not been well elucidated. The age at presentation varies between case series, with a 14-patient case series by Pagni et al. having an average age of 50.6 years, with no significant predilection for either gender [3]. TAMT may develop on an underlying diseased aorta due to underlying atheroma, aortic dissection, intimal flap, or aortitis [3, 5, 7, 9, 14]. Occasionally, TAMT may develop on what appears to be a normal underlying aorta. In this instance, thrombosis is most commonly due to a prothrombotic state, whether hereditary or acquired [10]. In addition, an underlying proinflammatory state associated with inflammatory bowel disease or systemic autoimmune collagen vascular disease such as rheumatoid arthritis has been reported as the underlying cause of thrombosis by Elder et al. [8] and Krishnamoorthy et al. [11], respectively. Despite exhaustive investigation, no cause could be identified in a strikingly large number of cases [3, 6, 12], as in this case.

The aim of therapy is to abolish the thrombus burden whilst ensuring no further embolism occurs. One therapeutic option is medical therapy alone, by means of anticoagulation, for example, using heparin and warfarin, with or without antiplatelet therapy, such as aspirin and clopidogrel. The second option is an interventional approach using adjunctive anticoagulation and either open surgical thrombectomy or TEVAR. Treatment of this rare entity has not been established or tested in case control trials or large meta-analyses. Many authors have reported resolution of clot and no further thromboembolism with anticoagulation with or without antiplatelet agents alone [3, 4, 6–8, 12, 13], while others have also had success with open surgical thrombectomy and postoperative anticoagulation [3, 6, 9, 10, 14], with the added benefit of addressing the organised thrombus component which is unlikely to respond to anticoagulation but at a risk of increased perioperative morbidity and mortality. A number of authors have, on the basis of trends in the literature, recommended early surgical over medical therapy. Pagni et al., in their review of 80 patients in case series from 1997 to 2010, found the recurrence or failure rate of open surgical intervention to be 16%, as compared to 24% for anticoagulation alone [3]. Although the numbers are small and statistically insignificant, it represents a strong trend favouring surgical treatment. It is noteworthy that the vast majority of case series and reports document only symptomatic aortic thrombus, that is, having resulted in embolic disease. The treatment consensus of asymptomatic thoracic aortic mobile thrombus is even more obscured.

Thoracic endovascular aortic repairs (TEVAR) using endovascularly deployed stents have found themselves into common use in treating non-TAMT aortic pathology with large studies supporting their use, having reduced early morbidity and mortality and equal late mortality compared with open surgery, yet at the cost of increased need for reintervention and follow-up [6, 15]. There are however few case reports of success with endovascular treatment of mobile aortic thrombus with exclusion of the aortic thrombus from the aortic lumen in the abdominal aorta [6, 16], with even fewer reporting success with thoracic thrombi [17], that is, TEVAR.

TEVAR was chosen in this case for three reasons. Firstly, it is the authors' opinion on review of the literature that anticoagulation therapy alone insufficiently addresses a possible organised thrombus component and has a theoretical risk of dissolving the thrombus stalk before the thrombus body, resulting in recurrent thromboembolic disease. Secondly, the patient was seen as a high risk for open surgery, and thus a less invasive approach seemed appropriate. Thirdly, it was felt that stent placement could occur successfully above T6 and thus any compromise of the artery of Adamkiewicz and spinal function would be less likely.

## 3. Conclusion

TAMT is a rare clinical entity usually diagnosed during cardioembolism workup and is complicated by major clinical embolic consequences. While there is no consensus on what constitutes optimal management, interventional approaches seem to be superior in terms of mortality and recurrence. It is to be borne out in future studies whether TEVAR offers a viable alternative in TAMT as it has in other aortic pathologies.

## Supplementary Material

Video 1: 2D TEE X-plane imaging demonstrating the mobile thrombi with no evidence of a dissection or overt atheromatous plagues.
Video 2: 3D TEE showing the mobile pedunculated thrombus at its attachment point to the aortic wall with no surrounding aortic atheroma or dissection visable in the proximal descending aorta.
Video 3: 3D TEE demonstrating three mobile discrete thrombi at the same level distal to the attachment point of the large pedunculated thrombus. The distal end of the latter thrombus is the large moble thrombus at approximately 10 ‘0 clock which can be seen to track up the aorta.
